# A Comprehensive Model of Audiovisual Perception: Both Percept and Temporal Dynamics

**DOI:** 10.1371/journal.pone.0023811

**Published:** 2011-08-22

**Authors:** Patricia Besson, Christophe Bourdin, Lionel Bringoux

**Affiliations:** Institute of Movement Sciences, CNRS - Université de la Méditerranée, Marseille, France; Kyushu University, Japan

## Abstract

The sparse information captured by the sensory systems is used by the brain to apprehend the environment, for example, to spatially locate the source of audiovisual stimuli. This is an ill-posed inverse problem whose inherent uncertainty can be solved by jointly processing the information, as well as introducing constraints during this process, on the way this multisensory information is handled. This process and its result - the percept - depend on the contextual conditions perception takes place in. To date, perception has been investigated and modeled on the basis of either one of two of its dimensions: the percept or the temporal dynamics of the process. Here, we extend our previously proposed audiovisual perception model to predict both these dimensions to capture the phenomenon as a whole. Starting from a behavioral analysis, we use a data-driven approach to elicit a Bayesian network which infers the different percepts and dynamics of the process. Context-specific independence analyses enable us to use the model's structure to directly explore how different contexts affect the way subjects handle the same available information. Hence, we establish that, while the percepts yielded by a unisensory stimulus or by the non-fusion of multisensory stimuli may be similar, they result from different processes, as shown by their differing temporal dynamics. Moreover, our model predicts the impact of bottom-up (stimulus driven) factors as well as of top-down factors (induced by instruction manipulation) on both the perception process and the percept itself.

## Introduction

Human beings need to efficiently collect information from their environment in order to make decisions about which action to perform next and to evaluate their actions' impact on this environment. They access this information through the perception process. This process can be understood as an inverse problem, where the cause (the physical source) must be identified from the observed stimuli. This problem is ill-posed since only partial and noisy information is conveyed by the senses [1], [2]. To arrive at a stable solution (a percept), some constraints based on high-level knowledge are used and modulate the way the information is used. Joint processing of the information collected by the different senses also constrains the perception problem, as it can help solve some ambiguities. Hence, perception can be seen as a system where complex processing of the sensory information is performed, working from the received stimuli (system inputs) to the percept itself (system output).

Several studies have addressed the question of understanding and modeling multisensory perception. Some focused on modeling how different input conditions (different spatio-temporal properties of the stimuli, or multisensory versus unisensory presentation of the information) yield different spatial [3]–[6] or temporal [7] percepts. Others investigated the impact of these different input conditions on the perception process itself from a temporal perspective, through the analysis of reaction times in detection tasks [8], [9] or in localization tasks [10]. The former studies aim at an understanding of how the outputs of the perception system are impacted by different contexts, whereas the latter aim at investigating the perception process itself - in particular, its dynamics. Though the results of these separate analyses suggest that the types of sensory stimulus or the mode of presentation impact both the perception process and its final output, no single model accounts for these two elements, and thus for the whole multisensory perception process.

In this paper, we propose a generative model of the perception process involved in a spatial localization task, in varying contexts, i.e., for different types of sensory stimulus (acoustic or visual) and for different modes of presentation (unisensory or multisensory). Our objective is not only to investigate and model the impact of these different contexts on the percepts (i.e. the outputs of the process), as in our previous work [11], [12], but to extend this to a comprehensive model accounting for the process itself. To this end, our new model embeds a temporal mark (the decision time) which characterizes the process dynamics. This comprehensive model therefore constitutes the added-value of the present paper with respect to both the state of the art and our previous work.

As far as the spatial percept - or output - is concerned, cross-modal biases occur when there is multisensory information. Most of the existing models resort to a Bayesian formalism to infer the output of the perception system [2], [13]. Indeed, Bayesian inference affords a principled and flexible statistical approach to inverse problems. It is particularly appropriate to model the perception process - which is inherently uncertain - since the constraints can be embedded straightforwardly in the form of prior probability distributions. Thus, the prior - on the way the information is handled - is assumed to be uniform in the classical maximum likelihood model (MLE) [5], [6], which explains the integration of multisensory information as a means for the brain to increase the reliability of the sensory estimates [5]. Indeed, as mentioned, multiple sources of information may help constrain the inverse problem by alleviating some ambiguities [1]. However, for stimuli showing specific physical properties, the multisensory biases may be very weak, or the information even segregated [2], [4], [11], [14]. Therefore, generalizations of the MLE model have recently been proposed, where non-uniform a priori are used, so that the two possible means of processing multisensory information (integration or segregation) are taken into account by the model [3], [4], [7], [11], [14], [15]. As specifically shown in our earlier work [11], the subjects exploited the available audiovisual information in different ways, depending on the type of sensory stimulus they were asked to locate (acoustic or visual). They integrated the audiovisual information when asked to locate the acoustic stimulus, whereas locating the visual stimulus was conditionally independent of the acoustic information. This confirms that, while multisensory information constrains the inverse problem, some higher-level constraints also play a part in the perception process. The Bayesian network (BN) we built earlier modeled the relationship structures connected with these two modes of multisensory information processing, as well as their dependence on the type of sensory stimulus, and ultimately inferred a spatial percept [11], [12].

The dynamics of the perception process have been widely explored, especially through analysis of reaction time. It has been established that multisensory information speeds up reaction times (multisensory enhancement) in both detection and localization tasks [10], [16], [17]. Brain level investigations using electroencephalography by humans [10], [16], [18] or animals [19] support the view that early stages of the perception process are involved, while late response stages are not significantly affected [10]. The reaction time to a primary stimulus can be shortened if an accessory - possibly irrelevant spatially - stimulus is presented at approximately the same time (intersensory facilitation of reaction time [20]). To the best of our knowledge, no behavioral model has been proposed as a support to the study of the dynamics of the perception process.

The generative model of the audiovisual perception process we present here yields both the percept from a spatial localization task and a temporal feature of the process dynamics. Our comprehensive model straightforwardly supports the statistical data analysis we first perform. In keeping with our earlier model [11], [12], we employ Bayesian networks and we focus on making the structure of the variable's statistical relationships emerge from the data throughout the model elicitation process. To this end, we use the information theoretic framework proposed in [11]. The structure of the relationships stresses the possible invariants attached to the perception process [21]. As such, it conveys more interesting information about the causal links between the subject's percept and the environment than the quantitative strengths of these links do. The model we propose is more general than the MLE model and is relevant to different contexts. First, the type of sensory stimulus to be located is either acoustic or visual. Secondly, both unisensory and multisensory information processes are studied, in the latter case producing cross-modal biases of varying strengths.

The paper starts with a brief reminder of our experimental protocol in [11] combining audiovisual perception with a spatial localization task. Both the subjects' spatial percepts and their decision times are then investigated. Then the relationships among variables are systematically analyzed and the model is elicited step by step. Finally, the results of the behavioral and of the Bayesian network analyses are discussed.

## Analysis

### Behavioral analysis

#### Experimental protocol

The procedure is briefly outlined here, the interested reader being referred to [11] for a more detailed description.

Ten subjects, seven males and three females (mean age 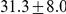) participated in the experiment. They were all right-handed with normal hearing and normal or corrected-to-normal vision. Informed written consent was obtained from all participants. Since only non-invasive behavioral measurements were carried out, the study was approved by the Institute of Movement Science Laboratory Review Board. The experiment was conducted in accordance with the Declaration of Helsinki.

The subjects were seated in complete darkness, in front of a curved screen. This screen bore nine red LEDs at equal distance and aligned in the azimuthal eye plane; it had a mobile buzzer above it. Two sessions, acoustic and visual, were performed in alternative order on two groups, each composed of half the subjects. In the acoustic perception task, a 35-ms-long acoustic stimulus (*primary stimulus*) was emitted at each trial, sometimes together with a visual stimulus (*secondary stimulus*), sometimes alone. The subjects were asked to report where they heard the sound. In the visual perception task, the primary and secondary stimuli are the visual and acoustic stimuli respectively. The subjects were asked to report where they saw the flash. The primary stimulus occurs randomly at 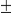
10 deg, 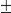
5 deg or 0 deg, and the secondary stimulus, when used, at 0 deg (coincident stimuli), 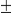
5 deg or 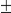
10 deg (non-coincident stimuli) from the primary stimulus position. Hence, possible positions for the secondary stimulus are 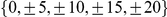.

To report the perceived location of the main stimulus, the subjects used a rotating pointer linked to a potentiometer. The subjects held the tip end and moved it from the right stop position of the pointer (the neutral position, located at 40 deg) to the perceived position. They remained in this position for about one second before coming back to the neutral position. They were free to move the pointer at the speed they wished.

The precise instructions given to the subjects were to locate the sound in the acoustic perception task, and the light in the visual perception task. They were informed that the acoustic stimulus might come with a visual stimulus in the acoustic perception task, and vice-versa in the visual perception task. Nevertheless, the instructions clearly asked them to focus on the primary modality.

For each task (acoustic or visual), the subjects were exposed to 450 stimuli: 75 unimodal stimuli (15 stimulus occurrences per position) and 375 bimodal stimuli. The latter include 75 spatially coincident stimuli (15 occurrences at each of the 5 possible primary stimulus positions) and 300 non-coincident stimuli (60 per primary stimulus position, 15 per secondary position). The whole data set thus comprises 4500 output values corresponding to the 10 subjects' responses to each input stimulus.

#### Output of the perception process

The perception process outputs are the subjects' spatial localizations of the primary stimuli. These were presented in [11], and we will only recall briefly some of the main results here. Our objective being to study and model multisensory perception, variations in the subjects' spatial responses that are unrelated to the percept itself must be removed as far as possible. Thus, a bias is observed in the subjects' answers, which is not significantly different between the acoustic and visual tasks for a given subject (for unisensory inputs), whereas it is between subjects. These inter-subject differences are then related to the sensorimotor component rather than to multisensory perception itself. They are smoothed by removing the mean of each subject's responses to unisensory stimuli, as was done in [11]. As a result, the normalized subjects' spatial localization - adopted hereunder - can be assumed to be an approximate observation of the subjects' spatial percept.

**Figure 1:**
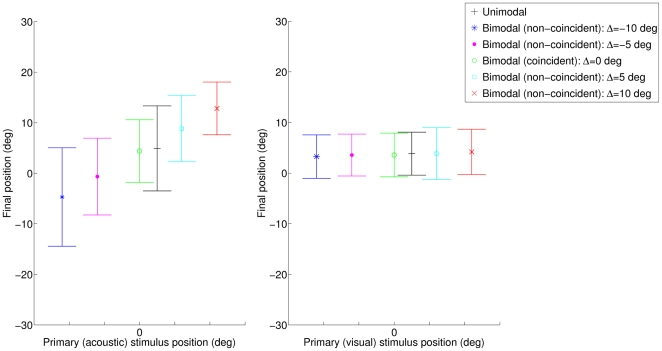
Means and standard deviations of the values indicated by the subjects when locating the median (0 deg) acoustic and visual stimuli in the unisensory, coincident and non-coincident cases. The values of the possible secondary stimuli are given as distances from the primary stimulus positions.

The mean and standard deviations of the system outputs (values indicated by the subjects) for primary stimuli occurring in the subjects' median plane (0 deg) are shown in Fig. 1. Confirming the visual dominance reported for spatial localization tasks [22], the subjects were more accurate and less variable (i.e. more precise) in the visual than in the acoustic perception task (averaged standard deviations equal to 7.5 deg and 2.8 deg respectively). Adding a spatially coincident secondary stimulus improved the precision of localization in the acoustic perception task (standard deviation equal to 5.8 deg), whereas it slightly decreased this precision in the visual perception task (standard deviation equal to 3.2 deg). Generally speaking, the subjects' localizations of the primary stimuli were strongly impacted by the secondary stimuli in the acoustic perception task, contrary to what happened in the visual perception task (for non-coincident stimuli, the standard deviations were 9.5 deg in the acoustic localization task, against 3.0 deg in the visual localization task).

#### Dynamics of the perception process

We now extend the analysis performed on our data set to take into account two temporal features, movement and decision times, both of them potentially related to the dynamics of the perception process. Movement onset is defined as the time when the pointer velocity exceeds 1.5 deg/s. Conversely, movement end is considered to be when it fell below 1.5 deg/s. This cutoff was chosen after careful data inspection and is comparable to values found in the literature (e.g., [23] after tangential velocity conversion). Decision time, which we distinguish from reaction time since there was no time constraint in our experiment, separates the presentation of the stimulus from movement onset. A statistical analysis of these features is now performed in order to establish whether one of them can be discarded.

A 2 tasks (visual vs acoustic)×3 modes of presentation (unisensory vs bisensory non-coincident vs bisensory coincident) Analysis of Variance (ANOVA) was conducted on the mean movement times recorded for target localization. It revealed neither significant main effects (Task: p = 0.934; Mode of presentation: p = 0.119) nor interactions between the two factors (p = 0.443). In other words, neither the nature of the stimuli, nor the uni- vs multisensory mode of presentation has any significant impact on movement time.

Since movement time is heavily dependent on motor characteristics, it is not surprising that it does not explicitly convey the dynamics of the perception process. Therefore, we normalized the movement time by the distance to be traveled in order to minimize this bias. A 2 tasks (visual vs acoustic)×3 modes of presentation (unisensory vs bisensory non-coincident vs bisensory coincident) ANOVA was performed on the mean movement time/distance to be traveled. As for movement time, this variable revealed neither significant main effects (Task: p = 0.946; Mode of presentation: p = 0.176) nor interactions between the two factors (p = 0.520).

**Figure 2:**
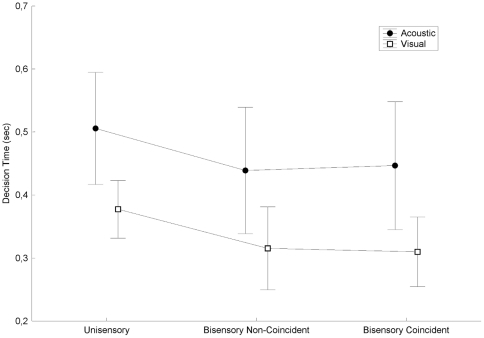
Decision time as a function of context (type of sensory stimulus and mode of presentation).

Finally, a 2 tasks (visual vs acoustic)×3 modes of presentation (unisensory vs bisensory non-coincident vs bisensory coincident) ANOVA was conducted on the mean decision time. Contrary to the two previous temporal indicators, it revealed a significant main effect of the task (F(1,9) = 8.983, p = 0.015) and of the mode of presentation (F(2,18) = 26.571, p = 0.000). As illustrated in Fig. 2, while visual stimuli are located more rapidly than acoustic stimuli, the mean decision time appears significantly shorter in bisensory than in unisensory presentations (Newmann-Keuls tests: unisensory vs bisensory coincident or non-coincident p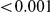; no difference between bisensory coincident and bisensory non-coincident).

To further explore the impact of the stimulus location, a 2 tasks (visual vs acoustic)×5 primary spatial locations ANOVA was conducted. In addition to the main task effect observed above (F(1,9) = 7.654, p = 0.022), the statistical analysis yielded a significant main effect of the primary stimulus location (F(4,36) = 5.502, p = 0.001). However, data inspection and post hoc tests showed that this effect was due to only one single target, whose eccentricity was maximal (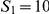 deg). Apart from this point, no obvious influence was found on decision time (Newman-Keuls tests showed no significant difference for other locations). The interaction between the two factors was not significant.

**Figure 3:**
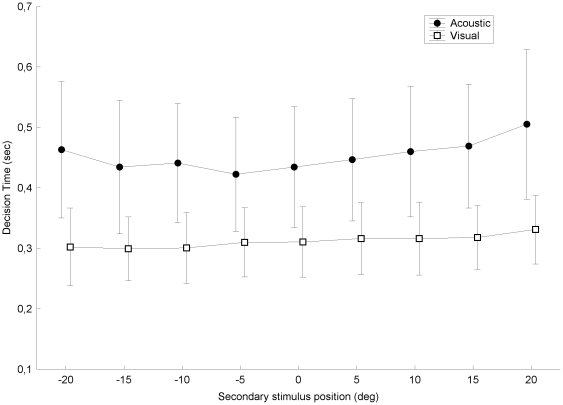
Decision time as a function of the secondary stimulus positions.

The influence of the secondary stimulus location was also investigated by a 2 tasks (visual vs. acoustic)×9 secondary spatial locations ANOVA. As previously, it revealed a main task effect (F(1,9) = 8.316, p = 0.018), but also a main effect of the secondary target location (F(8,72) = 5.966, p = 0.000) and a significant interaction between the two factors (F(8,72) = 2.822, p = 0.009). Post hoc tests and visual inspection of the data (see Fig. 3) confirm that decision times are longer in the acoustic than in the visual task, that no effect of the secondary stimulus location is found in the visual task (i.e., no secondary acoustic influence on decision time), and that effect of the secondary stimulus location in the acoustic task is marginal and mostly due to the most eccentric 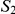 position (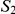 = 20 deg).

The first stage of our approach required us to identify the relevant variables to be embedded. As far as the percept itself was concerned, the choice was relatively straightforward. The normalized spatial positions indicated by the subjects approximately represent the percepts (since the task is spatial localization). Three temporal features, the subjects' movement times normalized or not by the distance to be traveled, and the subjects' decision times, were investigated in order to decide which better characterize the perception process. The statistical analysis we carried out showed that, unlike their movement times, the subjects' decision times were far more dependent on the sensory nature and on the mode of presentation of the stimuli than on their position. Therefore, decision time is deemed the temporal variable best characterizing the perception process.

### Bayesian network analysis

#### Statistical formulations

We have chosen to follow a probabilistic approach relying on Bayesian networks to model audiovisual perception, as there is an inherent uncertainty in the way the environment is perceived and processed by our sensory system. Specifically, a step-by-step elicitation of the model using BNs provides means of investigating the relationships among the variables involved in the perception process. To this end, we will use the information theoretic framework we proposed in [11]. Thus, we must cast the problem in a statistical framework to start with.

The primary and secondary stimuli are modeled by two random variables (rvs) 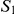 and 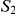 enumerating the possible stimulus positions, 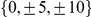 and 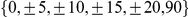 respectively. The last 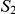 range value is arbitrarily assigned to the secondary stimulus in the unimodal case. The model yields two rvs, 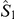 and 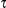. 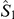 denotes the perceived primary stimulus localization and takes on values in the continuous range 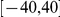, bounded by the physical limitations of the pointer. The second rv 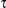 models the subjects' decision time. 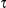 is defined on 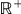. We also introduce two binomial rvs, 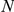 and 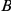. 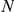 models the type of primary sensory stimulus. It is set to 0 in the acoustic perception task, and to 1 in the visual perception task. 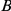 equals 0 if the inputs are unisensory, 1 if they are bisensory.

The rv probability density functions (pdfs) are estimated using histograms. The bin width to estimate the pdfs of the input signals 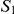 and 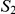 is set to five, and one bin is centered on each possible value of the ground truth (so that there are five bins in total for the primary stimuli, and ten bins for the secondary stimuli). This way, the ground truth pdfs are uniform. Moreover, any possible inaccuracy pertaining to the experimental design is taken into account. The range of 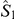 is covered by 15 bins: thirteen bins of width 5 are centered on 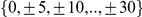 and two larger bins cover the bounding ranges 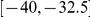 and 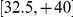, where the data is very sparse (hence a trade-off is maintained between the pdf estimate accuracy and overfitting). Obviously, the binomial pdfs of 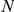 and 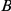 are estimated using two bin histograms, the bins being centered on 0 and 1. Finally, a histogram with 0.2 width bins centered on 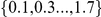 estimates the pdf of 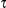. A 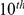 bin of no fixed width contains the few possibly remaining values of 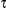. The histograms of 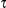 and 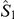 are provided in Figs. 10 and 11.

#### Model elicitation

First, the mutual information (MI) normalized by the joint entropy (so that direct comparisons can be performed) is estimated between pairwise rvs and compared to 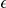 thresholds, to decide whether the values stand for dependent or independent variables. These thresholds allow for taking into account some possible approximations in the pdf estimates. Independent rvs are built by generating uniform pdfs on each rv's range. The MI values obtained with these artificial rvs give us the 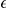 values. Then, conditional information (CMI) is computed to identify any third rv independences (see [11] for a detailed presentation of the method).

**Figure 4:**
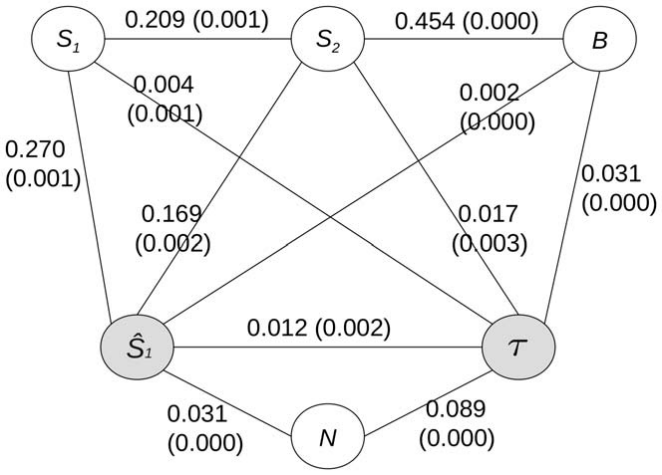
Undirected graphical model based on MI analysis. The shaded nodes are the model outputs. The MI values for to each edge are indicated with the corresponding 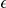 (thresholding) values (in brackets).

MI analysis yields the undirected structure presented in Fig. 4. As expected, 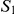 and 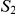 are independent of 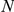 (the position of the stimuli are the same in the acoustic and visual perception tasks). Obviously, 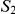 is largely dependent on 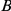 (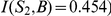 whereas 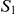 and 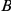 are totally independent. 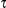 shows greatest dependence on 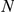 (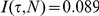), meaning that decision time is heavily affected by the type of primary sensory stimulus, as established in the data analysis section. 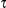 also depends strongly on 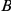 (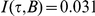): adding an accessory stimulus to the localization task impacts the decision time. Though weaker, the dependence between 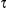 and the stimulus positions, 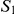 and 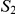, or the subject's stimulus localization 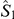 cannot be disregarded (the MI values are above their respective 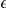 thresholds). In particular, the MI with 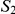 is only half as weak as 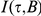 (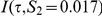).

**Figure 5:**
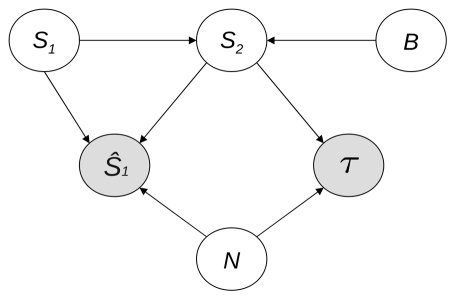
Graphical model 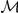 resulting from information flow analysis based on CMI values.

We now proceed to the CMI analysis, to identify potential third rv dependence. In pure machine learning problems, this step allows the inference computational cost to be decreased. In the present case, it reveals the causal relationships (in the causally sufficient senses as stated by Neapolitan in [24]) between environmental properties and the subjects' percepts. We observe that some CMI values are below their respective 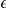 threshold values. An analysis of the information flow through the network (using the d-separation theorem [25]) leads to the partially directed acyclic graph [26] 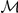 shown in Fig. 5. This analysis establishes that 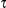 is conditionally independent of 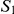 or 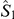. Contrary to what might be expected at first glance, 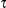 is also independent of 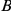 given 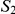 (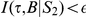). Actually, 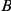 and 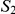 are largely redundant: they both follow Dirac distributions for unimodal inputs, but differ for bimodal inputs, where only 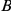 still follows a Dirac distribution. Thus, 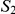, like 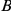, contains information about the existence or the absence of an accessory stimulus. But, contrary to 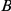, it also provides clues to the location of the information. Note that 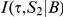 is also very small (0.004, which is equal to 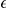) so that 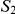 and 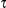 are not far from being conditionally independent given 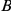. As a result, we can conclude that 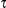 is primarily related to the mode of presentation (uni- or bisensory) of the incoming sensory information, although the position of the information is also a factor. Also, the model confirms the statement made in the previous section: decision time is conditionally independent of the primary input position 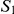, contrary to the percept 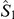, thus it primarily characterizes the perception process and not the pointing movement.

The probabilistic law described by 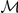 is:
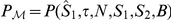


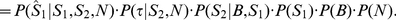
(1)Eq. (1) states that percept 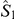 (output of the system) and decision time 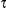 are conditionally independent given the context, i.e. given the sensory nature 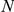 of the stimulus to be located, the position of the input stimulus, 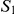 and 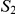, as well as whether or not there is a secondary stimulus 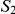 (implicitly, 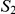 is the vector of the unisensory or bisensory property of the available information).

#### Context-specific independence

Since our aim was to bring to light specific top-down effects, depending on the context (environmental properties), we now focus on how changes in the environmental context modulate the structure of the variable relationships. To this end, we take our model-based analysis a step further by adding context-specific independence (CSI). CSI was formalized by Boutilier et al. in [27]. It is related to the so-called asymmetric independence used in similarity networks and multinets [28], [29]. While CMI reveals the possible structures of the relationship among variables for all the values these variables can take, CSI identifies dependences for different rv contextual values, i.e., for specific values of the rvs (note that we use the term *contextual value* rather than *context* as advocated in [27] to avoid any confusion with the previous utilization of the word *context* in the paper). Thus, CSI further generalizes Bayesian networks [30]. To represent the graphical network resulting from CSI analysis, we will resort to multinets, which allow CSI to be represented [31]. It is important to remember that when a context is assigned to a rv, the latter becomes a constant. As a result, its impact on the other graph variables is no longer captured by the graph structure. Instead, it is yielded by the quantitative expression of the joint probabilities described by the local networks of the multinet.

**Figure 6:**
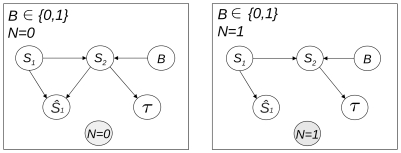
Local networks 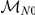 and 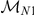 of the multinet 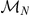, obtained for the respective contextual values 0 or 1 of 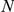.

Let us firstly assign the contextual values 0 or 1 to the rv 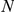. We obtain the multinet 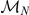 shown in Fig. 6, which reveals the structure of the variable relationships connected with the acoustic or visual localization tasks respectively. The structures of these local networks provide two interesting results. First, there are two different ways of handling the information as far as the percept is concerned, depending on the sensory nature of the stimulus to be localized. The percept is impacted by the accessory stimulus 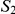 in the acoustic localization task (integration of the multisensory information), whereas it is conditionally independent of 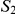 in the visual perception task (segregation of the multisensory information). Second, the structure of the relationships involving 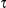 remains the same (the factors affecting the dynamics of the perception process are the same) whatever the type of primary sensory stimulus.

**Figure 7:**
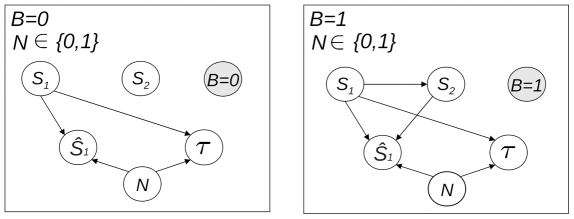
Local networks 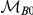 and 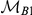 of the multinet 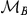 obtained for the respective contextual values 0 or 1 of 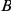.

We now remove any specific contextual value on 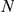 and set 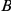 to 0 (unisensory inputs) or 1 (bisensory inputs). Analysis of the resulting dependences leads to the multinet 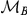 shown in Fig. 7. Both model outputs, 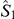 and 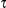, still depend on the type of sensory stimulus 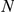, whatever the mode of presentation of the inputs. Unsurprisingly, with unimodal inputs, percept dependence on 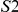 disappears, whereas it continues with bimodal inputs (let us remind that we are considering simultaneously the acoustic and visual localization tasks). Once the mode of presentation is fixed (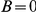 or 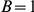), the dependence between 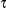 and 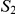 vanishes, to be replaced by a slight dependence between 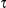 and 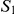. This confirms the hypothesis we put forward when we observed a conditional independence between 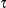 and 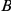 given 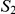: once 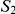 is no longer the vector of the mode of presentation, it only conveys (if it exists) clues about the spatial position of the information, as does 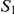.

## Results

While we are primarily concerned with eliciting the structure of the generative model, in order to investigate the structure of the implicit causal inference process attached to perception, we now examine the relevance of our model 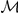, via a quantitative analysis. Note that if our objective had been to reduce the computational costs of inference, this quantitative analysis could have been done with the multinets 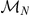 or 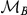, the joint pdf of the multinets being recovered via the union-product operator [32]. It needs to be seen whether the model is able to correctly infer the different percepts 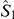 as well as the different decision times 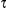, in relation to the multiple generated contexts.

Eq. (1) expresses the joint distribution 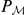 for the model 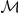 in terms of posterior and marginal distributions, whose parameters have to be learned. The posterior distributions are a Gaussian for 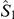 and a Log-normal for 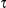 (i.e. taking the logarithmic values of the decision times yields normally distributed data). The conditional pdfs for 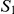 and 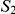 are uniform and are estimated by multinomial distributions.

A K-fold (with K = 10) cross-validation scheme is followed to learn the parameters and to perform the inference [33] so that no overlaps exist between the testing and the training sets. A maximum likelihood approach is used to learn the parameters of the multinomial and Gaussian distributions [34] on the training set. This training set is defined by the percepts and decision times of 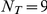 subjects randomly picked from the 10 subject set. Data for the remaining subject forms the testing set. Once the parameters of the pdfs have been learned, we perform inference (estimating the system outputs given the inputs) using a Maximum A posteriori (MAP) approach, where the MAP are defined as:
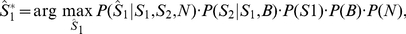
(2)

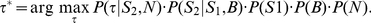
(3)Both the learning and inference stages were implemented using the Bayes Net Toolbox for Matlab [34].

**Figure 8:**
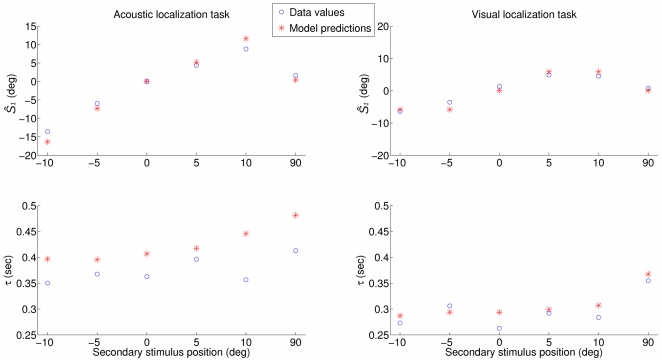
Observed and inferred outputs 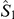 and 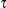 for the 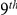 subject (training set consisting of all but this subject) when 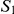 occurs at 0 deg, for 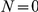 (left hand graph) and 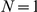 (right hand graph). We remind the reader that 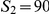 deg stands for the unimodal case.

We followed the training and testing procedure 10 times, on audiovisual uni- and bisensory data (with 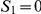 deg). The resulting mean coefficient of determination 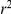 is 0.91 between the model's MAP and the subjects' percepts 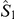, and 0.63 between the model's MAP and the subjects' decision times 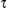. Table 1 details the mean 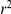 values obtained for the different position couples 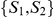. The model very well infers the subjects' percepts and fairly well infers the perception process dynamics attached to different secondary stimulus locations in different sensory and modal conditions. An example of the model's performance, when trained on all but the 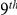 subject, then tested on this excluded 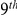 subject, is shown in Fig. 8, for the two contextual values of 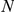 and for the different contextual values of 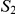 associated to the median position of 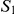 (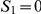 deg).

**Table 1:** Mean coefficients of determination between the data and the model.

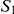 positions (in deg)	−10	−5	0	5	10
Mean 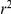 for 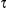	0.59	0.69	0.61	0.66	0.57
Mean 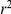 for 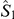	0.82	0.95	0.96	0.94	0.88

Mean coefficients of determination 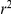 between the model predictions and the mean subjects' decision times 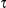 and localizations 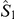, for different positions of 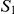 and 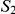 (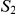 taking on values {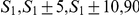}).

It can be observed from these plots that the model fully predicts the fusion and the non-fusion of the information that occurs in the acoustic and visual localization tasks respectively, while still correctly fitting the 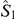 data in the unimodal case. It also quite faithfully infers the different decision times for the four possible contexts.

The lower 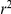 values for the decision times come certainly from the large inherent within-subject's variability. This impedes to get an accurate estimate of the mean subjects' decision time for each stimulus couples 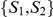. Actually, this result could be expected from the context-specific independence analysis we performed in the previous of the paper. Indeed, 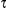 was shown to be conditionally independent of 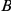 once 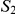 is known, because 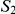 not only informs on the unisensory or bisensory property of the stimuli, but also pertains to variability in the subjects' answers. We ensured that this within-subjects' variability was not related to 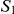 by testing a model where a direct link between 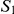 and 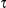 was present. The addition of this link did not improve the 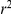 values. Increasing the number of presentations of the same stimulus couples 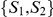 could theoretically solve the problem. Practically however, this would require a longer experiment where decrease of vigilance and increase of fatigue would certainly deteriorate the precision of the subject's answers.

**Figure 9:**
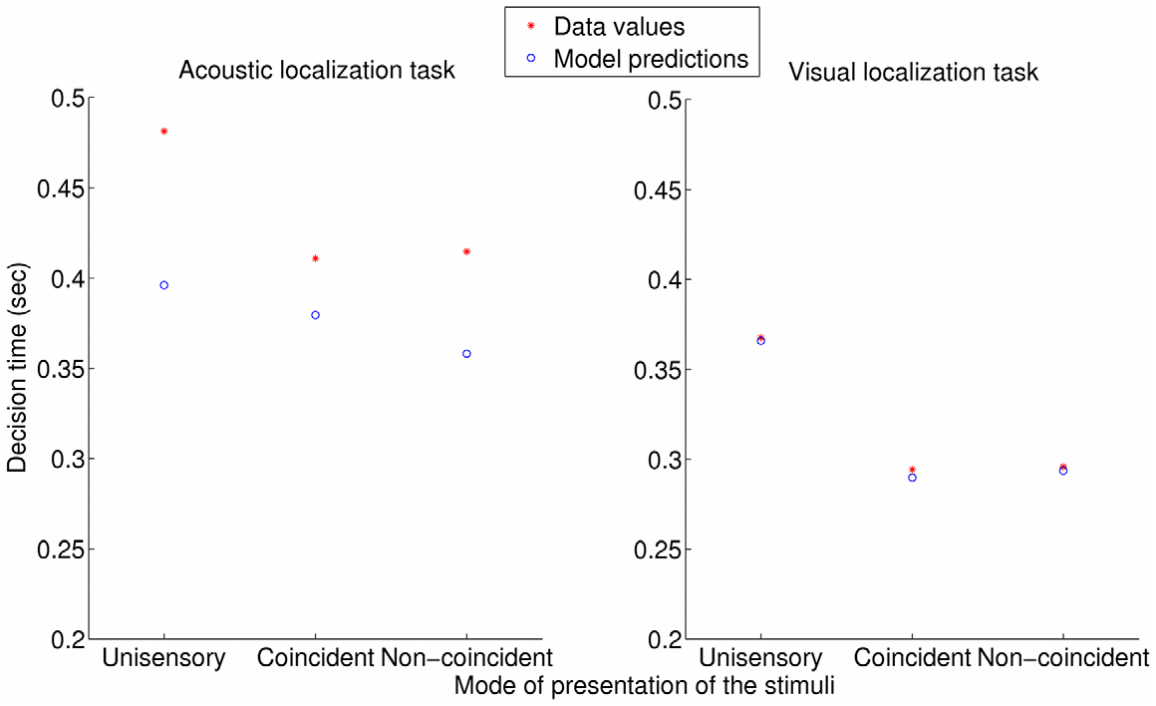
Observed and inferred outputs 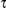 for the 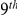 subject (training set consisting of all but this subject), for different contexts (type of sensory stimulus and mode of presentation).

Actually, the data analysis and the model's structure established that the decision times do not depend on 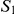 positions so that we can remove any reference to these positions and appraise the ability of the model to predict the subjects' decision times for different modes of presentation of the stimuli. This means that we now look at the data in a similar way than what was presented on Fig. 2. With the MAP values obtained for three specific positions of 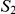 that correspond to three specific ways of presenting the information, namely, 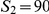 deg (unisensory case), 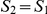 (bisensory coincident case) and 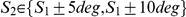 (bisensory non-coincident case), the mean coefficient of determination 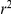 becomes 0.92. An illustrative example of these results is presented on Fig. 9, still for the 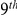 subject. Hence, when the secondary stimulus locations stand for different ways of presenting the information, the model is a very good predictor of the dynamics of the perception process.

**Figure 10:**
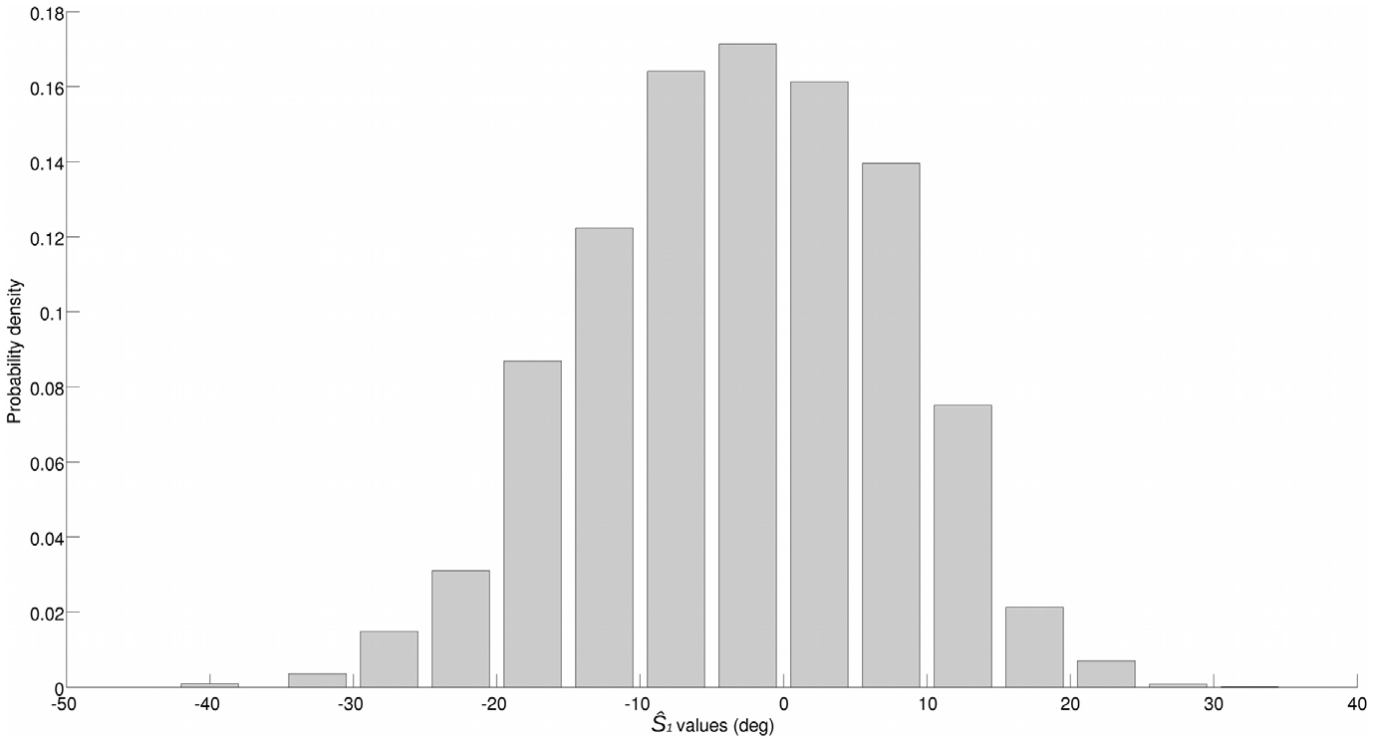
Probability density function of the subjects' spatial localizations 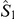.

**Figure 11:**
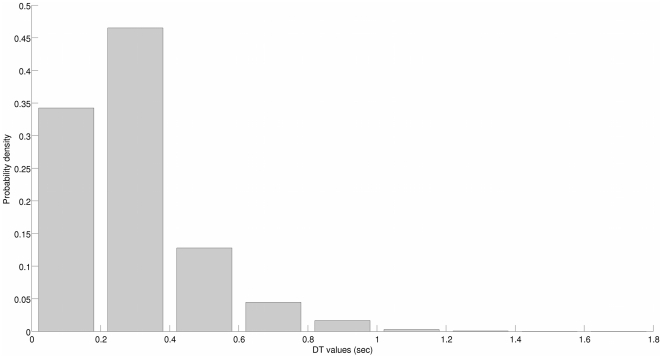
Probability density function of the subjects' decision times 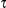.

## Discussion

This paper has addressed the question of understanding the perception process associated with an audiovisual localization task in its entirety. We do so by investigating and modeling not only the output of the process but also its temporal dynamics. The BN model that we propose, as a continuation and a formalization of behavioral analysis, meets this objective. The percept and the decision time, deemed to characterize the process dynamics, are both inferred for different environmental properties (contexts), i.e. for different types of sensory stimulus to be located, and for either unisensory or multisensory modes of presentation of the information. Our model is intended to investigate how multisensory integration is modulated by context, yielding different structural (bottom-up) and cognitive (top-down) factors.

To this end, our approach takes advantage of the compact representation of the problem domain offered by the BN structure, which depicts the relationships among the variables. Importantly, we made no a priori hypothesis about the model's structure, rather learning it from the data, following the information theoretic framework we proposed in [11]. To investigate the impact of different bottom-up and top-down factors on perception, we manipulated the context via observable variables that were then embedded in the model. Our data-driven approach thus differs from the ones taken in [4], [7], [35] where expert domain knowledge is used to hypothesize a model structure. In these models, a hidden variable mediates a model selection process, favoring either integration or segregation of the multisensory information. In our model, the observable variables 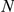 and 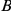 modulate the context and, as a result, the percept is shown to depend or not on both stimuli. 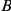 determines whether unisensory or multisensory stimuli are inputted (structural factor) while 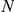 models the type of sensory stimulus to be located, i.e., it stands for instruction manipulation and, indirectly, for intention manipulation (cognitive factor).

As a result, the structure of the general model 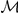 explicitly captures some of the data analysis results: the perception process does not only depend on certain structural properties of the stimuli, such as their spatial position and discrepancy, but also on other context properties that might induce top-down or bottom-up effects, such as the instructions given or the mode of presentation of the stimuli. To investigate this point further, we carried out a context-specific independence analysis of the relationships among the variables.

By assigning a contextual value to one of the variables, independences valid in this specific context only can be revealed. Thus, setting contextual values on 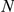 to specifically analyze the acoustic or the visual localization task yielded the local networks 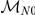 and 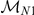 shown in Fig. 6. Unsurprisingly, they correspond partly to the models we proposed in [11], since 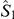 is conditionally independent of the secondary stimulus position 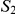 in the visual localization task (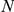 set to 1). As discussed in [11], this mathematically establishes that, in this case, the information is segregated at the percept level. The dominance of vision for spatial localization certainly explains this phenomenon. But the added value of the comprehensive modeling approach proposed here is that it reveals that the dynamics of the perception process are still dependent on whether the inputs are unisensory or multisensory (through the dependence on 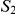), whatever the contextual value set for 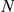. This establishes that multisensory integration is involved in both acoustic and visual localization tasks, even though in the visual context, percept 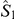 depends on the same input variables (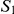 and 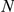) for both the multisensory and the unisensory cases.

Stated differently, the subjects receive and process multisensory information in both the acoustic and visual localization tasks, but they exploit this information differently depending on the sensory context. Therefore, as clearly shown by the global model we propose, multisensory integration phenomena (possibly reinforced by bottom-up cross-modal attention [36], [37]) constrain the inverse problem (e.g., shorter decision times yielding the same percept accuracy as in the unisensory case are observed in the visual perception task). However, prior knowledge and top-down processes (the instructions given to the subjects change their intentional or attentional focus) modulate the way this multisensory information is handled. Therefore, with multisensory inputs, there is an integration of the information - visible in the process dynamics - that results in a percept where this information is either fused or not.

Modeling approaches concerned with process output alone might miss this important result, which sheds light on the potentially wide variations in the underlying brain processes, depending on the bottom-up and top-down factors involved in the task. For example, the computational models proposed in [4], [7], [38] - or see [39] for a review - nicely predict the final percept associated with multisensory inputs characterized by different structural properties (mainly spatial congruency or discrepancy). However, these models cannot discriminate between similar outputs resulting from different processes. Similarly, while electrophysiological studies do investigate multisensory perception at the brain level, they limit their investigation to the temporal (dynamics) dimension of the process (see e.g. [10], [16], [18]).

On the basis of the present work, we are convinced that a comprehensive conceptualization of the perception process, where the output and the dynamics of the process are both investigated and modeled, should lead to a clearer understanding of multisensory perception. In particular, it should provide insights into the complex interconnections between perception and top-down factors, such as those induced by instruction manipulation for example. The latter may be related to intentional and attentional phenomena which closely interlock with multisensory integration, as discussed in [36], [37], [40]. Dedicated experimental protocols and joint behavioral and electrophysiological studies should be undertaken to investigate this point further.
